# Neuroprotective effect of an angiotensin receptor type 2 agonist following cerebral ischemia in vitro and in vivo

**DOI:** 10.1186/2040-7378-4-16

**Published:** 2012-08-24

**Authors:** Seyoung Lee, Vanessa H Brait, Thiruma V Arumugam, Megan A Evans, Hyun Ah Kim, Robert E Widdop, Grant R Drummond, Christopher G Sobey, Emma S Jones

**Affiliations:** 1 Department of Pharmacology, Monash University, Clayton, VIC, 3800, Australia; 2 School of Biomedical Sciences, The University of Queensland, Brisbane, QLD, 4072, Australia

**Keywords:** Apoptosis, AT2 receptor, Cerebral ischemia, Glucose deprivation, Mouse, Neuronal death, Neuroprotection, Stroke

## Abstract

**Background:**

Intracerebral administration of the angiotensin II type 2 receptor (AT_2_R) agonist, CGP42112, is neuroprotective in a rat model of ischemic stroke. To explore further its possible cellular target(s) and therapeutic utility, we firstly examined whether CGP42112 may exert direct protective effects on primary neurons following glucose deprivation *in vitro*. Secondly, we tested whether CGP42112 is effective when administered systemically in a mouse model of cerebral ischemia.

**Methods:**

Primary cortical neurons were cultured from E17 C57Bl6 mouse embryos for 9 d, exposed to glucose deprivation for 24 h alone or with drug treatments, and percent cell survival assessed using trypan blue exclusion. Ischemic stroke was induced in adult male C57Bl6 mice by middle cerebral artery occlusion for 30 min, followed by reperfusion for 23.5 h. Neurological assessment was performed and then mice were euthanized and infarct and edema volume were analysed.

**Results:**

During glucose deprivation, CGP42112 (1x10^-8^ M and 1x10^-7^ M) reduced cell death by ~30%, an effect that was prevented by the AT_2_R antagonist, PD123319 (1x10^-6^ M). Neuroprotection by CGP42112 was lost at a higher concentration (1x10^-6^ M) but was unmasked by co-application with the AT_1_R antagonist, candesartan (1x10^-7^ M). By contrast, Compound 21 (1x10^-8^ M to 1x10^-6^ M), a second AT_2_R agonist, had no effect on neuronal survival. Mice treated with CGP42112 (1 mg/kg i.p.) after cerebral ischemia had improved functional outcomes over vehicle-treated mice as well as reduced total and cortical infarct volumes.

**Conclusions:**

These results indicate that CGP42112 can directly protect neurons from ischemia-like injury *in vitro* via activation of AT_2_Rs, an effect opposed by AT_1_R activation at high concentrations. Furthermore, systemic administration of CGP42112 can reduce functional deficits and infarct volume following cerebral ischemia *in vivo*.

## Introduction

Stroke is the world’s second most common cause of death and a leading cause of long-term disability in adults [1]. Currently, there are very few safe and effective treatment options available for patients following stroke [2] and hence there is a great need to develop new therapies that may improve stroke outcome. It is conceivable that targeting elements of the renin-angiotensin system (RAS) may provide neuroprotection prior to and/or following stroke [3-11].

The major peptide of the RAS is angiotensin II (Ang II), which acts with equal affinity at two membrane-bound receptors, the angiotensin type 1 receptor (AT_1_R) and the type 2 receptor (AT_2_R). It is well established that excessive stimulation of the AT_1_R by Ang II mediates biologically detrimental actions in the setting of cerebrovascular disease [3,12-14], whereas activation of the AT_2_R may at least partly offset the effects of AT_1_R stimulation and is associated with a protective function [15]. In addition, blockade of the AT_1_R may be protective at least in part because it results in greater binding to and activation of the AT_2_R by Ang II [3-5]. A number of clinical trials have demonstrated the beneficial effects of sartan class drugs (AT_1_R antagonists) such as candesartan and eprosartan, given prior to and following stroke [16,17].

The AT_2_R is the predominant angiotensin receptor subtype expressed in fetal organs, and its expression is downregulated following birth [9,18]. Interestingly, recent studies have shown that under pathological conditions such as ischemic insult, AT_2_R expression may be upregulated [7,9] and it may play an important role in the growth, repair and regeneration of neuronal tissue.

To date only one study has tested for a neuroprotective effect of direct AT_2_R activation using a selective AT_2_R agonist in the setting of stroke, and it suggested that direct AT_2_R activation is beneficial during ischemic stroke in spontaneously hypertensive rats subjected to cerebral ischemia [8]. Specifically, intracerebral administration of the AT_2_R agonist, CGP42112, prior to ischemia resulted in a reduced infarct volume at 72 h [8]. This protective effect of CGP42112 was abolished by administration of PD123319 (an AT_2_R antagonist), confirming a role for AT_2_R. In the brain, AT_2_Rs are known to be expressed in neurons [7] and also in vascular cells [5], although the target cell types for the protective action of CGP42112 remain unknown.

In the present study, we firstly tested the hypothesis that selective AT_2_R activation is directly neuroprotective *in vitro* using primary cortical neuron cultures deprived of glucose. Secondly, we tested whether CGP42112 may be effective in limiting neurological deficit when administered systemically following cerebral ischemia in a mouse model of stroke.

## Materials and methods

### Animals

All animal experiments were performed in accordance with the National Health and Medical Research Council of Australia guidelines, and with approval from the Monash University Animal Ethics Committee (Projects SOBSB/2010/34 and SOBSB/2009/55). Thirteen pregnant C57BL6/J (Monash Animal Research Platform) females were used to obtain cortical neurons from E17 pups. For cerebral ischemia-reperfusion (I/R) experiments in vivo, we used a total of 40 male C57BL/6 mice (8-12 weeks old; weight = 26 ± 6g). Five mice were excluded from the study when, during the surgical procedure to induce cerebral ischemia-reperfusion: (1) there was inadequate (<70%) reduction in regional cerebral blood flow (rCBF) (n = 2); or (2) no measured increase in blood flow at reperfusion after 30 min ischemia (n = 1); or (3) animals died before 24 h of reperfusion had elapsed (n = 2).

### Primary neuronal cultures

Mouse primary cortical neurons were prepared as previously described [19] with some modifications. Timed pregnant mice were anesthetized using inhaled isoflurane and an incision was made in the abdominal wall to remove embryos, which were immediately placed and dissected in Hank’s balanced salt solution (HBSS) (Invitrogen, Melbourne) without Ca^2+^ and Mg^2+^, supplemented with HEPES (10 mmol/L) (Invitrogen, Melbourne), gentamicin (5mg/L) (Invitrogen, Melbourne). Dissected cortices (free of meninges) were digested in trypsin (1 mg/ml) (Sigma, Sydney) for 10 min at room temperature, neutralized with trypsin inhibitor (Sigma, Sydney) for 10 min, and washed three times with Neurobasal medium (NBM) (Invitrogen, Melbourne) supplemented with L-glutamine (2 mmol/L), gentamycin (5 mg/L), and B-27 supplements (Invitrogen, Melbourne) pH 7.2. Dissociated cell suspensions were resuspended in NBM (+ supplements) then dispensed into poly-D-lysine (Sigma, Sydney) coated 60-mm^2^ Petri dishes. Cells were incubated overnight at 37 °C in a humidified atmosphere of 5% CO_2_ in air. Medium was replaced with fresh NBM (+ supplements), and the cells were maintained for a further 8 d without renewal of the medium.

### Glucose deprivation

Glucose deprivation was used to induce slow cell death through apoptotic mechanisms, analogous to the post-ischemic neuronal death that occurs in the penumbra region surrounding an infarct core *in vivo*[20-22]. For glucose deprivation, cultured neurons (9 d *in vitro*) were incubated in glucose-free Locke’s medium containing (in mmol/L) NaCl 154.0, KCl 5.6, CaCl_2_2H_2_O 2.3, MgCl_2_6H_2_O 1.0, NaHCO_3_ 3.6, HEPES 5.0, pH 7.2, supplemented with gentamicin (5 mg/L) at 37 °C in a humidified atmosphere of 5% CO_2_ in air for 24 h. For drug treatments, cells were exposed to one or more of the following: CGP42112 (1x10^-8^, 1x10^-7^ or 1x10^-6^ M; GLS Biochem, Shanghai); PD123319 (1x10^-6^ M; Sigma, Sydney); Compound 21 (1x10^-8^, 1x10^-7^ or 1x10^-6^ M; kindly provided by A. Hallberg, Department of Medicinal Chemistry, Uppsala University, Sweden); or candesartan (1x10^-7^ M; Astrazeneca, Sweden). All drugs were dissolved and diluted in distilled water.

### Cell viability assay

Cell viability was determined by trypan blue exclusion. In brief, cells were incubated at 37°C in 0.2% trypan blue for 15 min. After washing three times with phosphate buffered saline (PBS; pH 7.4), cells were fixed with 4% paraformaldehyde for 10 min. Twenty images of random fields at 200 magnification were taken from each dish using an inverted microscope and camera (Nikon, Japan) and associated computer software NIS-elements version 3.0 (Nikon, Japan). Approximately one thousand cells were counted from each dish. Cell viability was determined based on colour, size and cellular morphology by an experimenter blinded to the treatment group, and the cell death occurring during the 24 h glucose deprivation period was calculated.

### Cerebral ischemia-reperfusion in vivo

We used a model of focal cerebral ischemia-reperfusion similar to that described previously [23,24]. Briefly, mice were anesthetized with a mixture of ketamine (80 mg/kg i.p.) and xylazine (10 mg/kg i.p.). A midline neck incision was made, and the right external carotid (ECA) and pterygopalatine arteries were isolated and cauterized. The internal carotid artery (ICA) was lifted and occluded at the peripheral site of the bifurcation of the ICA. Focal cerebral ischemia was induced by intraluminal filament occlusion for 30 min of the right middle cerebral artery (MCA) with a 6-0 nylon monofilament with a silicone-coated tip (0.20-0.22 mm, Doccol Co., Redlands, CA, USA). Severe (~75%) reduction in rCBF was confirmed using trans-cranial laser-Doppler flowmetry (Perimed) in the area of cerebral cortex supplied by the MCA (~2 mm posterior and ~5 mm lateral to bregma). Mice were treated i.p. with either vehicle (saline; n = 16) or CGP42112 (1 mg/kg; n = 19) at the commencement of reperfusion.

### Neurological assessment and quantification of infarct volume

Neurological deficit was evaluated using a five-point scoring system (0, no deficit; 1, failure to extend right paw; 2, circling to the right; 3, falling to the right; and 4, unable to walk spontaneously) and hanging wire test in a blinded fashion, as described previously [23-26]. Briefly, mice were suspended from a 30 cm high wire by their forelimbs for up to 60 s. Average hanging time (i.e. latency to fall) of 3 trials with 5 min rest in between was recorded. Cerebral infarct distribution and volumes of infarct and edema were also estimated as described previously [23,24].

### Double-label fluorescent immunohistochemistry

Serial coronal sections (10 μm thick) were collected for analysis. Brain sections were fixed in acetone for 15 min and washed in 0.01 M phosphate-buffered saline (PBS, 3 x10 min). Sections were blocked with 10% goat serum (Abcam) for 30 min then incubated overnight in anti-rabbit active Caspase 3 (1:200; Abcam) and anti-mouse NeuN (1:1000; Chemicon). Following several washes in PBS (3 x 10 min), sections were incubated with Alexa 594-conjugated anti-rabbit IgG (1:500; invitrogen) for 2 h in room temperature. Brain sections were then washed with PBS (3 x 5 min) and incubated in M.O.M Biotinylated anti-mouse IgG Reagent (Vector Laboratories) for 10 min. Sections were then again washed in PBS (2 x 2 min) and Fluorescein Avidin DCS (Vector Laboratories) was applied for 5 min. Sections were washed in PBS (3 x 10 min) and cover-slipped. Staining was analysed on an Olympus fluorescence/light microscope (Olympus, Hamburg, Germany).

### Data analysis

Results are reported as the mean ± standard error (SEM). Statistical differences (P < 0.05) were determined by one-way analysis of variance (ANOVA) with Dunnett’s post-hoc test or an unpaired *t*-test, as appropriate, using GraphPad Prism version 5 (Graph Pad Software Inc., San Diego, CA).

## Results

### Effects of AT_2_R agonists on death of primary cortical neurons during glucose deprivation

**Figure 1:**
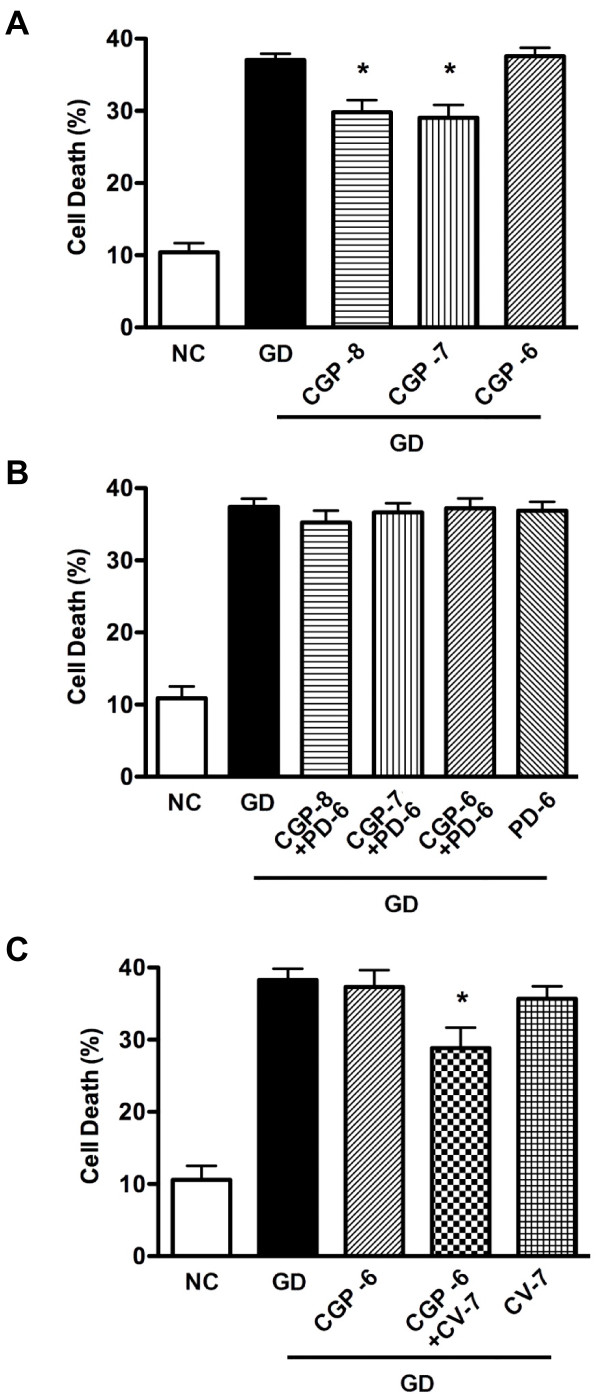
**Mean percentage neuronal cell death.A**. Data are shown for cells exposed to normal conditions (NC) or glucose deprivation with vehicle for 24 h (GD). Other cells exposed to GD were treated with CGP42112 at 1x10^-8^ M (CGP-8), 1x10^-7^ M (CGP-7), 1x10^-6^ M (CGP-6) (n = 8) (*P < 0.05 vs. NC). **B**. Data are shown for cells exposed to normal conditions (i.e. 24 h NC) or glucose deprivation with vehicle (24 h GD) or 3 concentrations of CGP42112 in the presence of PD123319 at 1x10^-6^ M (PD-6). Data for GD with PD123319 alone (n = 6) are also shown (*P < 0.05 vs. vehicle; NS P > 0.05 vs. vehicle). **C**. Data are shown for cells exposed to normal conditions (NC) or glucose deprivation (GD) with vehicle (24 h GD) or 1x10^-6^ M CGP42112 (CGP-6) in the absence or presence of 1x10^-7^ M candesartan (CV-7) (n = 7; *P < 0.05 vs. vehicle).

**Figure 2:**
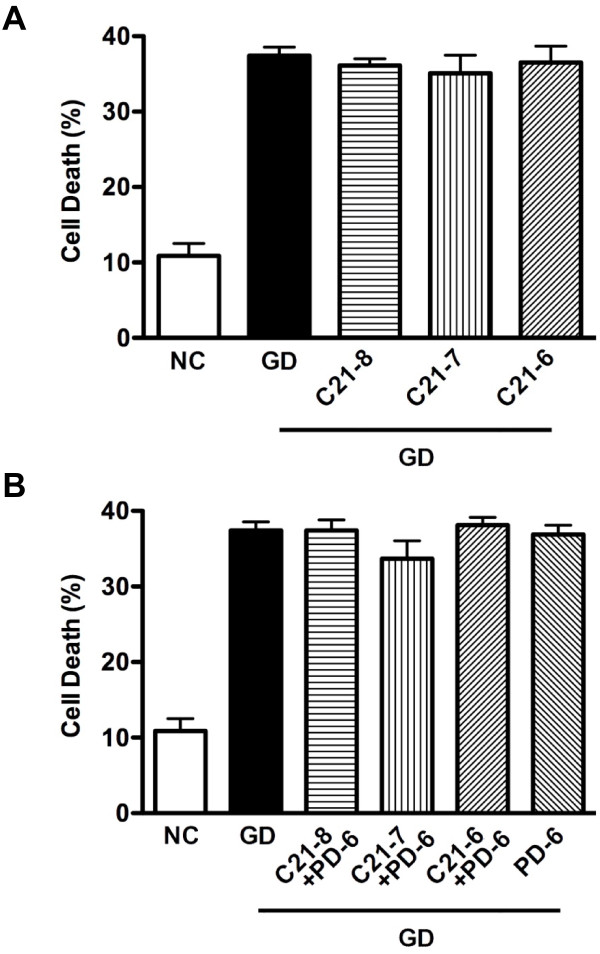
**Mean percentage neuronal cell death.A**. Data are shown for cells exposed to normal conditions (NC) or glucose deprivation with vehicle for 24 h (GD). Other cells exposed to GD were treated with Compound 21 at 1x10^-8^ M (C21-8), 1x10^-7^ M (C21-7), 1x10^-6^ M (C21-6) (n = 6) (NS *P* > 0.05 vs. vehicle). **B**. Cells were treated with Compound 21 in the presence of 1x10^-6^ M PD123319 (PD-6) (n = 6) (NS *P* > 0.05 vs. vehicle).

**Figure 3:**
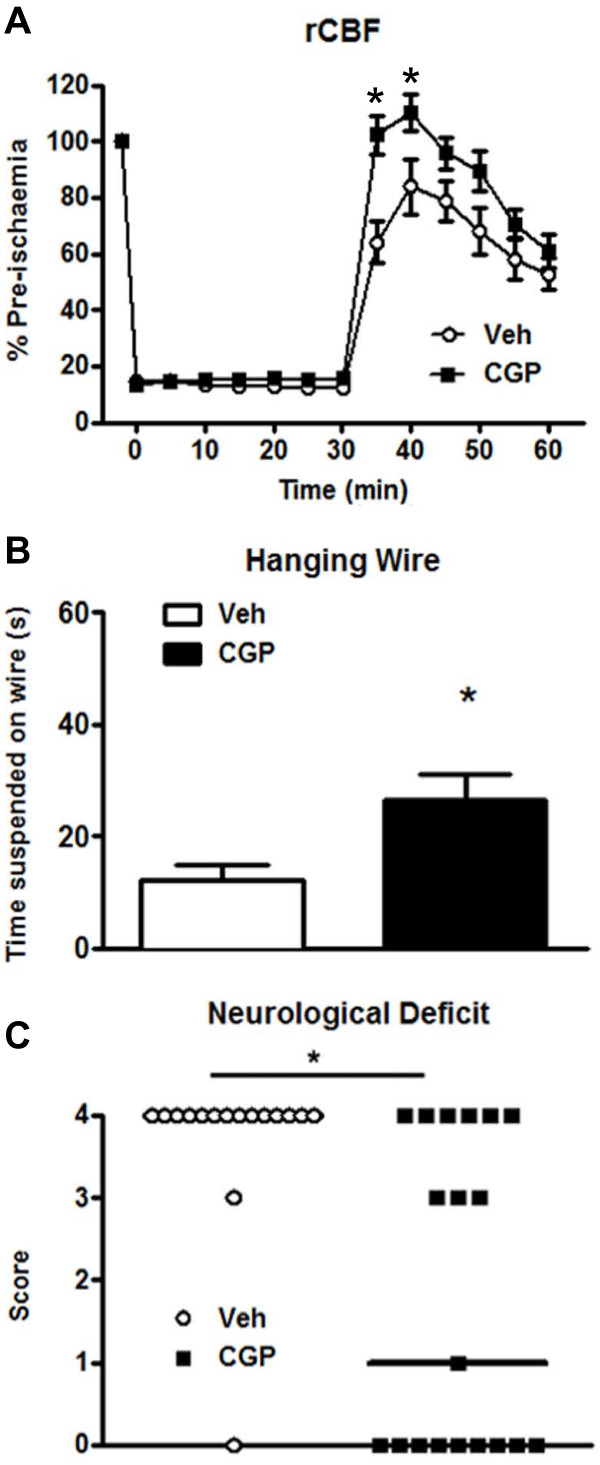
**Regional cerebral blood flow (rCBF) and neurological function in vehicle- and CGP42112-treated mice.** Data for rCBF (**A**), neurological deficit score (**B**) and hanging-wire test (**C**) are shown for animals 24 h following cerebral ischemia and treatment with either vehicle (Veh; n = 16) or CGP42112 (n = 19; *P < 0.05 vs. vehicle).

Following exposure of primary cortical neurons to 24 h of glucose deprivation, cell death was markedly enhanced (~35-40%; see Figures 1, 2, 3). Treatment with the AT_2_R agonist CGP42112 (1x10^-8^ or 1x10^-7^ M) resulted in a ~30% reduction in cell death due to glucose deprivation, whereas a higher concentration of CGP42112 (1x10^-6^ M) had no protective effect (Figure 1A). The protection by 1x10^-8^ M and 1x10^-7^ M CGP42112 was prevented by co-treatment with the AT_2_R antagonist PD123319 (1x10^-6^ M; Figure 1B). Protection by 1x10^-6^ M CGP42112 was unmasked by co-treatment with the AT_1_R antagonist candesartan (1x10^-7^ M; Figure 1C). In contrast to the effect of CGP42112, the putative AT_2_R agonist Compound 21 (1x10^-8^ to 1x10^-6^ M) exerted no effects on cell death induced by glucose deprivation (Figure 2A-B).

### Effect of systemic administration of CGP42112 on outcome following ischemic stroke

**Figure 4:**
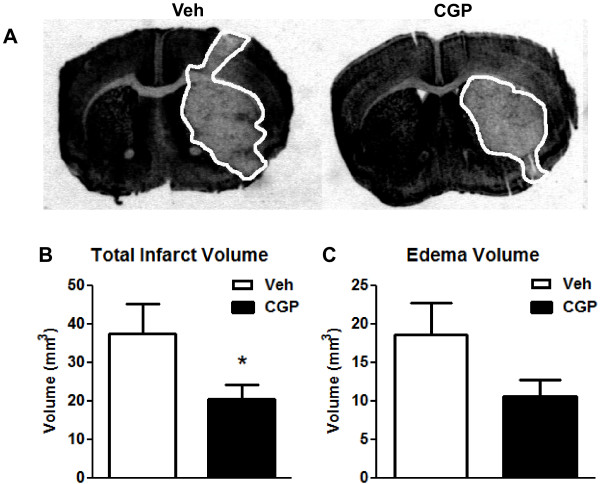
Total brain infarct and edema volume in vehicle- (Veh; n = 16) and CGP42112-treated mice (n = 19; *P **< 0.05 vs. vehicle).A**. Images of representative coronal brain sections from vehicle- (left) and CGP42112-treated (right) mice 24 h after cerebral ischemia. Total infarct volume (**B**) and brain edema volume (**C**).

**Figure 5:**
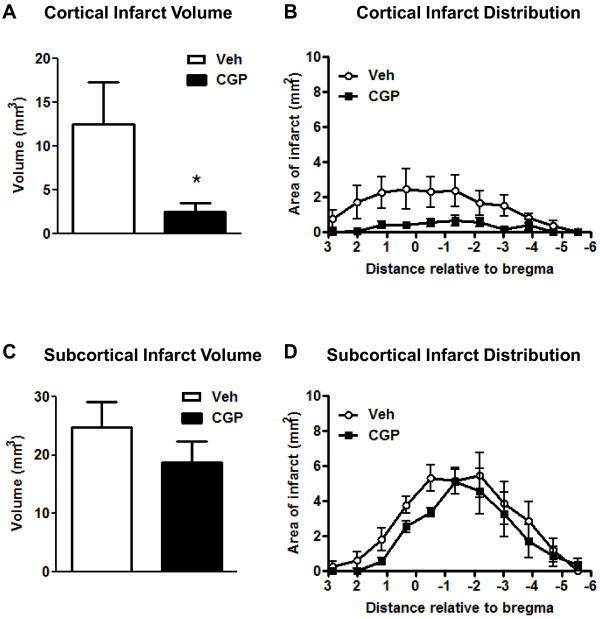
Cortical (A-B) and subcortical (C-D) brain infarct volume and distribution in vehicle- (Veh; n = 16) and CGP42112-treated mice (n = 19; *P **< 0.05 vs. vehicle).**

Vehicle or CGP42112 was injected at the time of reperfusion in all mice. Cerebral blood flow profiles were similar between the two groups of mice during the 30 min of middle cerebral artery occlusion and by the end of the 30 min recording period following reperfusion (Figure 3A). However, compared with vehicle treatment, blood flow was transiently but significantly higher in CGP42112-treated mice during the first 10 min of reperfusion (Figure 3A). Mice treated with CGP42112 also exhibited a significant improvement in functional outcomes at 24 h as measured using a neurological deficit score and by hanging wire time (Figure 3B-C). In addition, total and cortical infarct volumes in mice treated with CGP42112 were significantly reduced compared with vehicle-treated mice. (Figures 4 and 5). Edema volume also tended to be smaller in CGP42112- than vehicle-treated mice (Figure 4D).

### Effect of CGP42112 on neuronal apoptosis in vivo

Finally, we performed a small number of studies (4 vehicle-treated and 4 CGP42112-treated mice) to assess whether the neuroprotective effects of CGP42112 following stroke may be associated with attenuated neuronal apoptosis *in vivo*. For this, we utilised double-labelling immunohistochemistry for cells staining positive for cleaved/activated caspase 3 and NeuN (the neuronal marker). In each mouse brain, we counted between 600 and 3600 cells in 9 sections of somatosensory cortex (bregma +1.18 to -2.18 mm) at 200x magnification. Cleaved/activated Caspase 3 immunoreactivity was present in at least some sections from each of the vehicle-treated mice, at a frequency of 3.43 ± 1.03% of the 6,154 NeuN-positive cells counted. By contrast, no immunoreactivity for cleaved caspase 3 was detectable in any of the 36 sections, containing 5,709 NeuN-positive cells counted from CGP42112-treated mice (i.e. 0.00 ± 0.00%; p = 0.06 versus vehicle, n = 4, Wilcoxon signed rank test, GraphPad Prism). These preliminary data suggest that the post-stroke protection afforded by CGP42112 *in vivo* is associated with reduced neuronal apoptosis.

## Discussion

There are three new major findings of the present study. First, CGP42112, an AT_2_R agonist, exerts direct protective effects on primary neurons in culture to reduce cell death following exposure to glucose deprivation, an effect which was blocked by an AT_2_R antagonist, PD123319. Second, at a high concentration of CGP42112 (1x10^-6^ M), its protective effects are lost due to additional activation of AT_1_R, and this effect can be prevented with the AT_1_R antagonist, candesartan. Third, systemic administration of CGP42112 at the time of reperfusion following cerebral ischemia in mice results in substantially less neurological deficit and infarct volume at 24 h. Together, these results suggest that the neuronal AT_2_R is a valid therapeutic target for treatment following ischemic stroke and that CGP42112 can be administered systemically following stroke to bring about functional benefits.

We have previously reported that pretreatment with CGP42112 can limit infarct volume following cerebral ischemia in spontaneously hypertensive rats when injected intracerebrally into the region to be subjected to ischemia [8]. It is unknown what cell type(s) might be the target for such protective effects of CGP42112 *in vivo*. The use of primary neuronal cultures in this study has thus enabled us to evaluate whether CGP42112 might potentially have a direct protective effect on neurons subjected to ischemia-like conditions. Indeed, we found that this AT_2_R agonist (at 1x10^-8^ M and 1x10^-7^ M) can modestly protect neurons from such cell death. Our findings are analogous to those of Li et al. (2005) [7] whereby AT_2_R stimulation with Ang II significantly increased viability of cultured neurons subjected to glutamate-induced neuronal injury.

Interestingly, the neuroprotective effect of CGP42112 was lost at the highest concentration tested (i.e. 1x10^-6^ M). Incomplete selectivity of CGP42112 for the AT_2_R over the AT_1_R has been noted at high concentrations [27]. There is good evidence that activation of the AT_1_R may contribute to the pathology of stroke [15], as well as glutamate-induced neuronal injury [7]. We found evidence that the activation of neuronal AT_1_R by 1x10^-6^ M CGP42112 overides its AT_2_R-dependent neuroprotection, in that this high concentration of CGP42112 was protective if co-administered with the AT_1_R antagonist, candesartan. Such data support the notion that therapeutic effectiveness of AT_2_R agonists may be greater when co-administered with an AT_1_R antagonist [15].

Unlike CGP42112, we found no evidence for any protective effect by a similar range of concentrations of Compound 21. This drug, which is reported to be an orally active non-peptidic AT_2_R agonist, has been shown to have ~10-fold lower affinity than CGP42112 at the AT_2_R [28]. It is therefore possible that higher concentrations may have been required in the present study to sufficiently activate AT_2_R to provide protection.

Systemic administration of CGP42112, commencing at reperfusion, resulted in a markedly improved functional outcome and reduced infarct volume, particularly in the cortical region at 24 h following transient cerebral ischemia in mice. This finding is analogous to the above-mentioned study of McCarthy et al. who found that intracerebral injection of CGP42112 prior to cerebral ischemia exerted neuroprotective effects in the cortex [8]. The fact that systemic administration of CGP42112 achieved significant functional benefits associated with reductions in infarct volume and neuronal apoptosis suggests that movement of the drug across the blood-brain barrier was effective. Thus, the present work advances the concept that administration of an AT_2_R agonist at the time of tissue plasminogen activator-induced reperfusion is plausible as a combination treatment in stroke patients with successful post-ischemic reperfusion therapy. Further studies are required to test whether an AT_2_R agonist might also be protective following permanent cerebral ischemia (i.e. without reperfusion). Interestingly, CGP42112 treatment had no significant effect on infarct at subcortical regions or edema volumes at 24 h. It is possible that the clear beneficial effects of CGP42112 on functional outcome measures include facilitation of neuronal activity [7,29] as well as simply preventing neuronal death. Future studies to clarify the extent of protection by CGP42112 treatment at later timepoints and also at different doses are warranted.

While our results suggest that AT_2_R located on neurons could mediate protective effects of CGP42112 in ischemia-like conditions, there are a number of other cell types which may participate in the AT_2_R-mediated neuroprotection observed *in vivo*. In particular, the AT_2_R located on endothelial cells could play an important role in AT_2_R-mediated neuroprotection following stroke by supporting cerebral blood flow via endothelium-dependent vasodilatation [30]. Consistent with this possibility, we found that blood flow velocity in the cortical region impacted by cerebral artery occlusion was transiently higher early during reperfusion in animals treated with CGP42112. It remains to be determined whether non-neuronal brain cells, such as astrocytes and oligodendrocytes, might also express AT_2_R that contribute to improved functional outcome following CGP42112 treatment [31]. Furthermore, Iwanami *et al.* (2011) have recently demonstrated that AT_2_R located on haematopoietic cells may be a target for achieving neuroprotection following stroke [6]. Elucidating the contribution of multiple cell types involved in AT_2_R-mediated neuroprotection would aid in a better understanding of the neuroprotective potential of AT_2_R agonists *in vivo*.

It is important to note that we cannot fully exclude the possibility that infarct volume was still not fully developed at 24 h in this study, and thus it is possible that the protective effect of CGP42112 after stroke *in vivo* represented a delay in injury development rather than a complete prevention of it. While we have previously found that infarct volume is no greater at 72 h than at 24 h after 30 min of middle cerebral artery occlusion in C57Bl6 mice [23], a definitive conclusion regarding the sustained protection by CGP42112 cannot be made without a longer timepoint being studied.

In summary, the present study provides evidence that AT_2_R activation by the agonist CGP42112 can both directly protect against neuronal cell death following glucose deprivation *in vitro*, and improve functional outcomes in association with reduced infarct volume when administered systemically following cerebral ischemia-reperfusion *in vivo*. Overall, the findings support the hypothesis that administration of an AT_2_R agonist could be a useful adjunct in the clinical treatment of acute stroke.

## Abbreviations

Ang II: Angiotensin II; AT_1_R: Angiotensin type 1 receptor; AT_2_R: Angiotensin type 2 receptor; MCA: Middle cerebral artery; RAS: Renin-angiotensin system; ECA: External carotid artery; ICA: Internal carotid artery; rCBF: Regional cerebral blood flow.

## Competing interests

The authors declare that they have no competing interest.

## Authors’ contributions

SL performed animal and cell culture experiments, data analysis and contributed to writing the manuscript. VHB and HK performed animal experiments. MAE performed data analysis. TVA, GRD, REW, ESJ and CGS designed the study, helped to interpret data and contributed to the manuscript. All authors read and approved the final manuscript.
